# The GluN2A Subunit Regulates Neuronal NMDA receptor-Induced Microglia-Neuron Physical Interactions

**DOI:** 10.1038/s41598-018-19205-4

**Published:** 2018-01-16

**Authors:** Ukpong B. Eyo, Ashley Bispo, Junting Liu, Sruchika Sabu, Rong Wu, Victoria L. DiBona, Jiaying Zheng, Madhuvika Murugan, Huaye Zhang, Yamei Tang, Long-Jun Wu

**Affiliations:** 1 Department of Neurology, Mayo Clinic, Rochester, MN 55905 USA; 2 Department of Cell Biology and Neuroscience, Rutgers University, Piscataway, NJ 08854 USA; 3 Department of Neurology, Sun Yat-Sen Memorial Hospital, Sun Yat-Sen University, Guangzhou, 510120 China; 4 Department of Neuroscience and Cell Biology, Rutgers Robert Wood Johnson Medical School, Piscataway, NJ 08854 USA

## Abstract

Microglia are known to engage in physical interactions with neurons. However, our understanding of the detailed mechanistic regulation of microglia-neuron interactions is incomplete. Here, using high resolution two photon imaging, we investigated the regulation of NMDA receptor-induced microglia-neuron physical interactions. We found that the GluN2A inhibitor NVPAAM007, but not the GluN2B inhibitor ifenprodil, blocked the occurrence of these interactions. Consistent with the well-known developmental regulation of the GluN2A subunit, these interactions are absent in neonatal tissues. Furthermore, consistent with a preferential synaptic localization of GluN2A subunits, there is a differential sensitivity of their occurrence between denser (*stratum radiatum*) and less dense (*stratum pyramidale*) synaptic sub-regions of the CA1. Finally, consistent with differentially expressed GluN2A subunits in the CA1 and DG areas of the hippocampus, these interactions could not be elicited in the DG despite robust microglial chemotactic capabilities. Together, these results enhance our understanding of the mechanistic regulation of NMDA receptor-dependent microglia-neuronal physical interactions phenomena by the GluN2A subunit that may be relevant in the mammalian brain during heightened glutamatergic neurotransmission such as epilepsy and ischemic stroke.

## Introduction

Microglia are resident immune cells in the central nervous system (CNS) and their functions have become appreciated in normal physiology from development through adulthood. Microglial involvement in CNS physiology ranges from the regulation of neural circuit development in the immature brain^1–3^ to neurogenesis and learning in the adult brain^4–7^. Furthermore, microglial activities have been implicated during CNS dysfunction in diseases as well as in normal aging^8–10^. These observations suggest that microglia and neurons engage in a delicate balance of intercellular communication that needs to be carefully regulated^1,11–13^. Indeed, microglia are the most dynamic resident cells of the CNS and make transient physical contact with neuronal boutons, dendrites and somata^14–18^. Moreover, excitatory and inhibitory neurotransmission have been shown to reciprocally modulate this microglial surveillance^19^. However, our understanding of the repertoire of microglia-neuron physical interactions and its underlying mechanisms are not exhaustive.

Microglia have also now been suggested to regulate neurotransmission. For example, genetically perturbing microglial fractalkine receptors during development alters neurotransmission^20,21^ that could have long lasting effects into adulthood^22^. Moreover, selectively activating microglia enhances excitatory neurotransmission^23^. In this light, microglia are integrated into neuronal synapse function leading to a further revision of the synapse model into the “quad-partite” model^24,25^. Recently, we and others^17,18,26^ were able to uncover physical microglia-neuron interaction axes that occur during intense glutamatergic activation of neuronal NMDA receptors (NMDAR). We found that NMDAR activation triggers purine release that elicits microglial process extension (MPEs) and microglial process convergence (MPCs) through P2Y12 receptors^17,18^. In the current study, we continued these investigations to provide further insights into the mechanisms showing the requirement for the GluN2A NMDAR subunit in these microglia-neuron physical interactions.

## Results

### Regulation of NMDAR-Induced Microglia-Neuron Physical Interactions by the GluN2A Subunit

We and others recently reported that glutamate activation of neuronal NMDARs elicits MPEs through microglial P2Y12 receptors^18,26^. To provide further insights into its underlying mechanisms, we investigated the role of specific subunits of the NMDAR on MPEs using a pharmacological approach. NMDARs are composed of several subunits including obligatory GluN1 and GluN2 subunits—the GluN2A and GluN2B. With a wealth of interest in the role of the GluN2A and GluN2B subunits in NMDAR function in the CNS^27,28^, we investigated the potential subtype-dependent regulation of microglia-neuron interactions by NMDAR activation. To this end, we used Ifenprodil (“ifen”, 3 µM), a well-recognized GluN2B antagonist, and NVPAAM007 (“NVP”; 0.4 µM), a GluN2A antagonist^29^.

**Figure 1:**
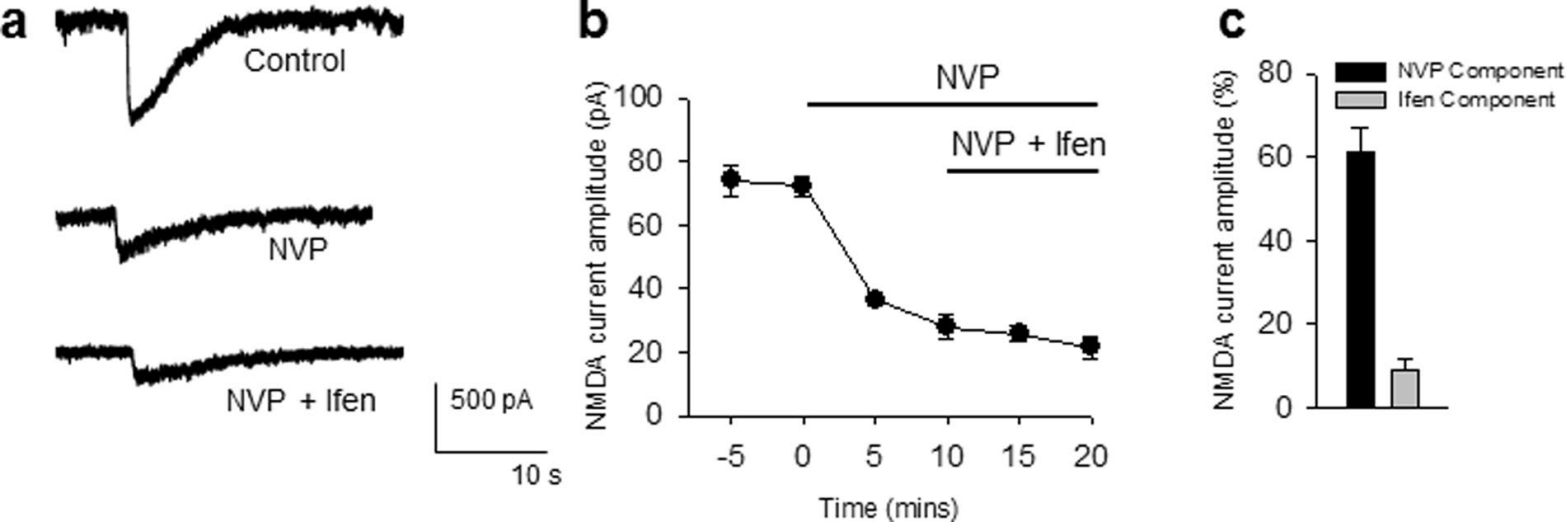
GluN2A/B Subunit Contributions to NMDAR-induced Currents. (**a**) Sample tracings of NMDA(100 µM)-induced currents under basal conditions (top), following 10 min of NVPAAM007 (0.4 µM, middle) and combined NVPAAM007 and Ifenprodil (3 µM, bottom). (**b**) Graph of NMDA-induced current amplitude with applications of GluN2 antagonist. (**c**) Graph showing the average percent of NVP- and ifenprodil-sensitive components of the NMDA-induced current after sequential application of NVP and ifenprodil. All data are presented as mean ± S.E.M. n = 3 slices each.

**Figure 2:**
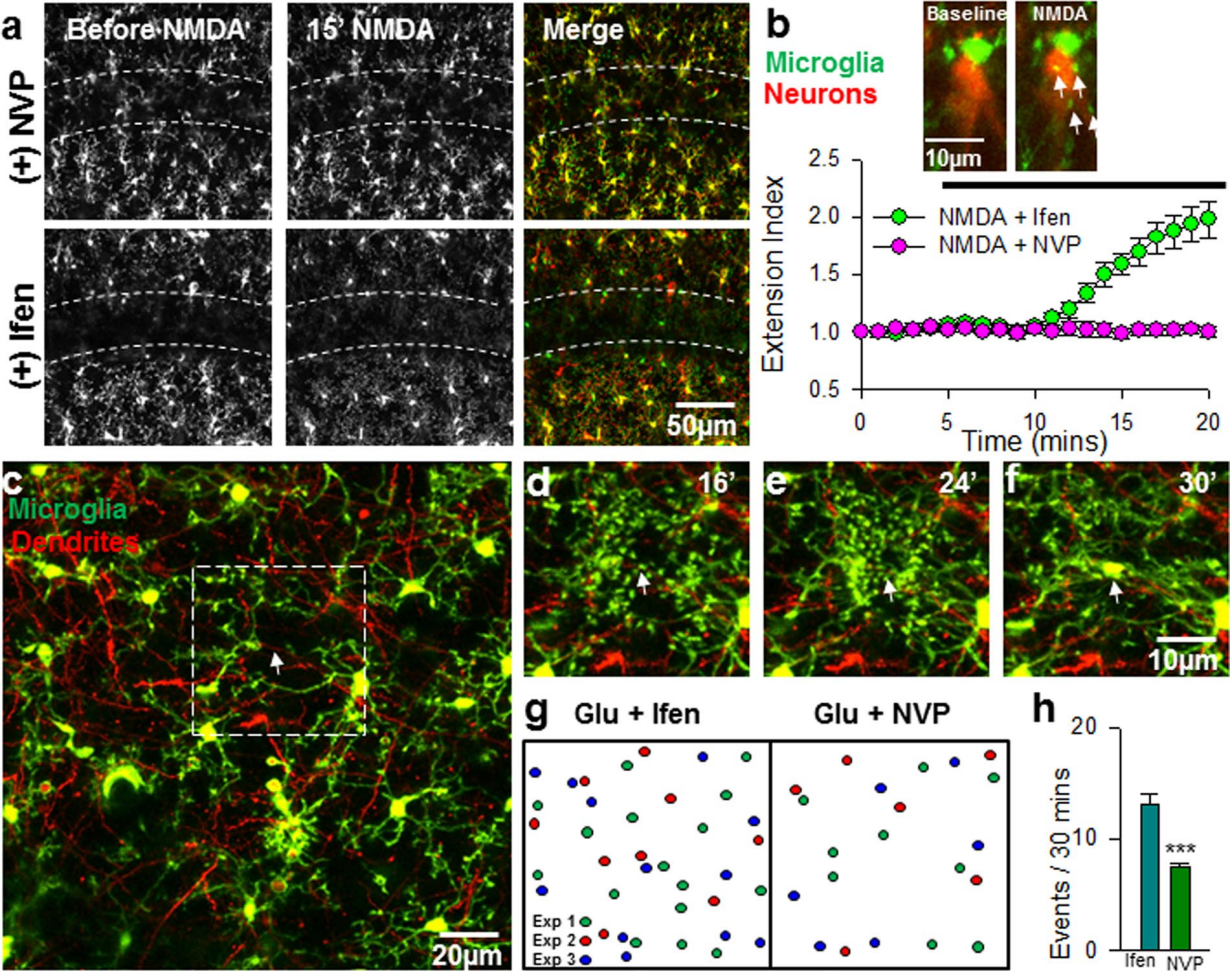
Regulation of NMDAR-induced Microglia-Neuron Physical Interactions by the GluN2A Subunit. (**a**) Representative z-stack two-photon images of GFP-expressing microglia in hippocampal CA1 of acute brain slices before (left) and after 15 min of NMDA (30 µM) treatment (center) in the presence of GluN2A antagonist (top) or GluN2B antagonist (bottom). Rightmost images are merged images of the before (red) and after (green) images. Extending microglial processes can be visualized in the *stratum pyramidale* layer (dashed lines) in green. **(b**) Quantitative summary of corresponding data to (**a**); insert: high magnification image collected from a double trangenic mouse brain slices following NMDA-treatment. The images show microglial processes (GFP-labelled) making bulbous tipped contact (white arrows) with a neuron (YFP-labelled) following NMDA treatment. (**c–f**) Representative image of the field of view with boxed region that is expanded to show timelapse images of converging microglial processes (green) that terminate on a neuronal dendrite (white arrow) that occurs after NMDAR activation. (**g,h**) A schematic of sites of microglial process convergence events during a 30 minute imaging period from three representative experiments (**g**) and quantified summary (**h**) showing significantly reduced events with NVP but not Ifen during NMDA treatment. All data are presented as mean ± S.E.M. n = 4–6 slices each. ***P < 0.001.

We first tested for GluN2A and GluN2B components of the NMDA-induced currents in pyramidal neurons using these drugs. Consistent with our previous results^29^, we show that NVP blocks a majority (~60%) of the NMDA-induced current amplitude while ifen further blocked about 10% of the NMDA-induced current (Fig. 1a-c). Pre-incubation with either drug did not alter basal microglial motile dynamics (data not shown). As expected, the perfusion of NMDA (30 µM, 15 min) induced robust microglial process extensions (MPEs) to hippocampal CA1 area (Fig. 2a,b). Interestingly, co-application of NMDA with NVP but not ifen abolished NMDAR-induced MPEs (Fig. 2a,b; see also Supplementary Video 1). This indicates significant roles for the GluN2A subunit of the NMDAR in MPEs.

We recently reported the existence of another form of microglia-neuron physical interaction which we termed microglial process convergence (MPCs)^30^, a phenomenon that was observed in epileptic conditions in an NMDAR-dependent manner^17^. Though distinct from MPEs, we speculated that MPCs are also regulated by the GluN2A subunit. Consistent with this hypothesis, compared to ifen, NVP significantly reduced the occurrence of MPCs following a 10 minute glutamate (1 mM) treatment (Fig. 2c–h). Together, these results indicate that the GluN2A subunit regulates NMDAR-induced microglia-neuron physical interactions including both MPEs and MPCs.

### Developmental Regulation of NMDAR-Induced Microglia-Neuron Physical Interactions

**Figure 3:**
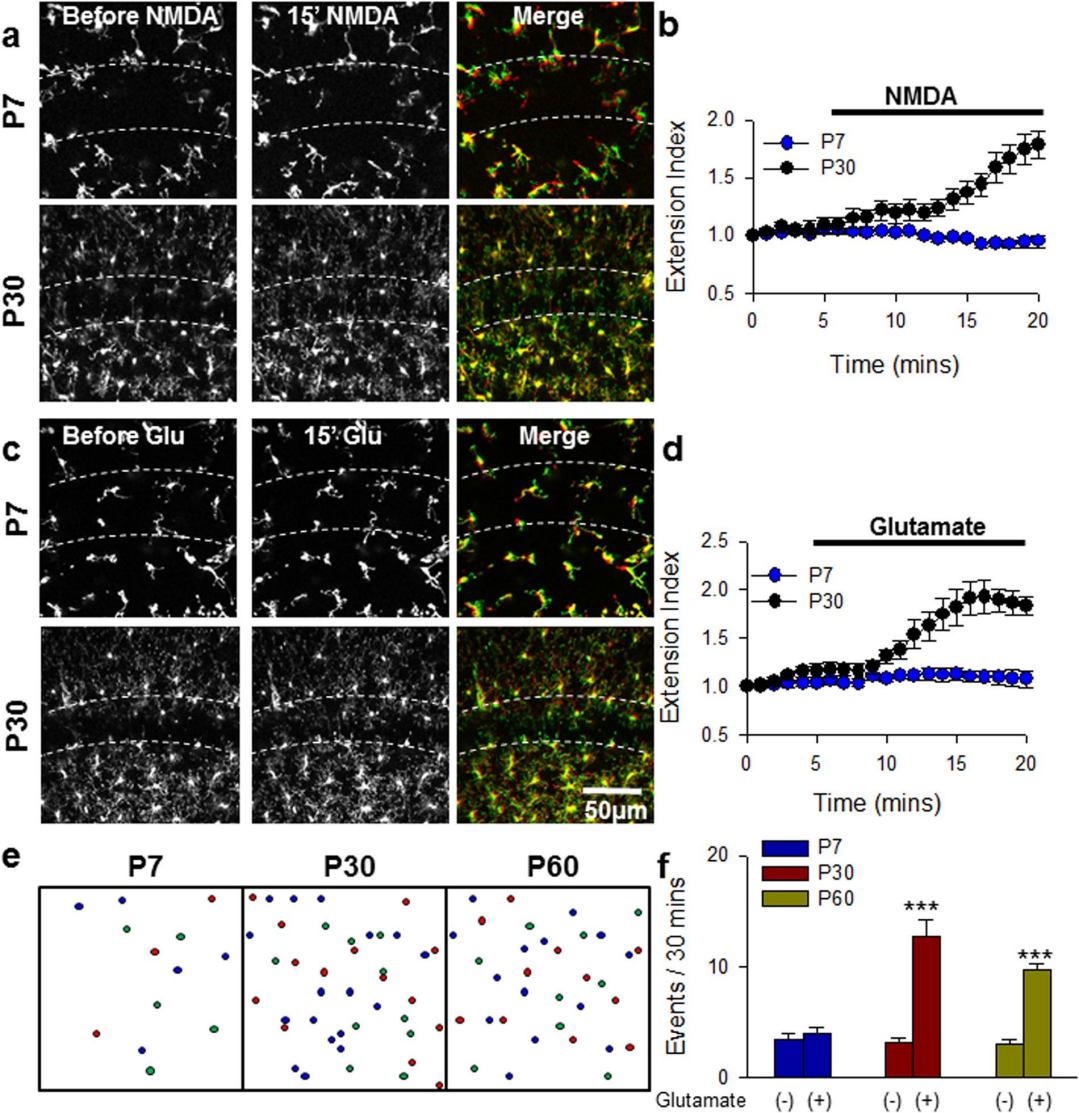
Developmental Regulation of NMDAR-induced Microglia-Neuron Physical Interactions. **(a–d**) Representative z-stack two-photon images of GFP-expressing microglia in hippocampal CA1 of acute brain slices before (left) and after 15 min of NMDA (**a**, 30 µM) or glutamate (**c**, 1 mM) treatment (center) in a P7 slice (top) or P30 slice (bottom). Rightmost images are merged images of the before (red) and after (green) images. Extending microglial processes can be visualized in the *stratum pyramidale* layer (dashed lines) in green. Quantitative summary of corresponding data to (**a**) and (**c**) are provided in (**b**) and (**d**), respectively. (**e,f)**, Schematic showing microglial process convergence event sites (**e**) and summary (**f**). Results indicate the failure of glutamate to increased microglial process convergence events in P7 but not P30 or P60 slices. All data are presented as mean ± S.E.M. n = 4–6 slices each. ***P < 0.001.

GluN2A and GluN2B subunits exhibit a differential developmental regulation such that GluN2B predominate during early postnatal development which eventually gives way to the later predomination of GluN2A subunits during latter postnatal development into adulthood^31–33^. Considering that the GluN2A but not the GluN2B subunit is required for MPEs, we investigated the possibility of a developmental regulation of MPEs. Interestingly, we found that NMDA (30 µM) failed to induce MPEs in P7 tissues although we could observe the phenomena in tissues from P30 mice (Fig. 3a,b; see also Supplementary Video 2) and even as early as P12 (data not shown). Similarly, glutamate (1 mM), the physiological agonist of NMDARs, failed to elicit MPEs in P7 tissues though it induced robust MPEs in P30 tissues (Fig. 3c,d; see also Supplementary Video 3). Likewise, MPCs did not occur in response to glutamate treatment in slices from P7 mice while a robust occurrence was observed in P30 and P60 brain slices (Fig. 3e,f). Together, these results indicate that there is a developmental regulation of NMDAR-induced microglia-neuron physical interactions that is consistent with the well-documented developmental switch from predominant GluN2B expression during early postnatal development to later predominant GluN2A expression. For simplicity and given the similarities in the GluN2A and developmental regulation of both MPEs and MPCs, we focused on MPEs for the rest of our studies.

### Differential Sensitivity to NMDAR-Induced Microglial Process Extension in the *Stratum Radiatum* and *Stratum Pyramidale*

**Figure 4:**
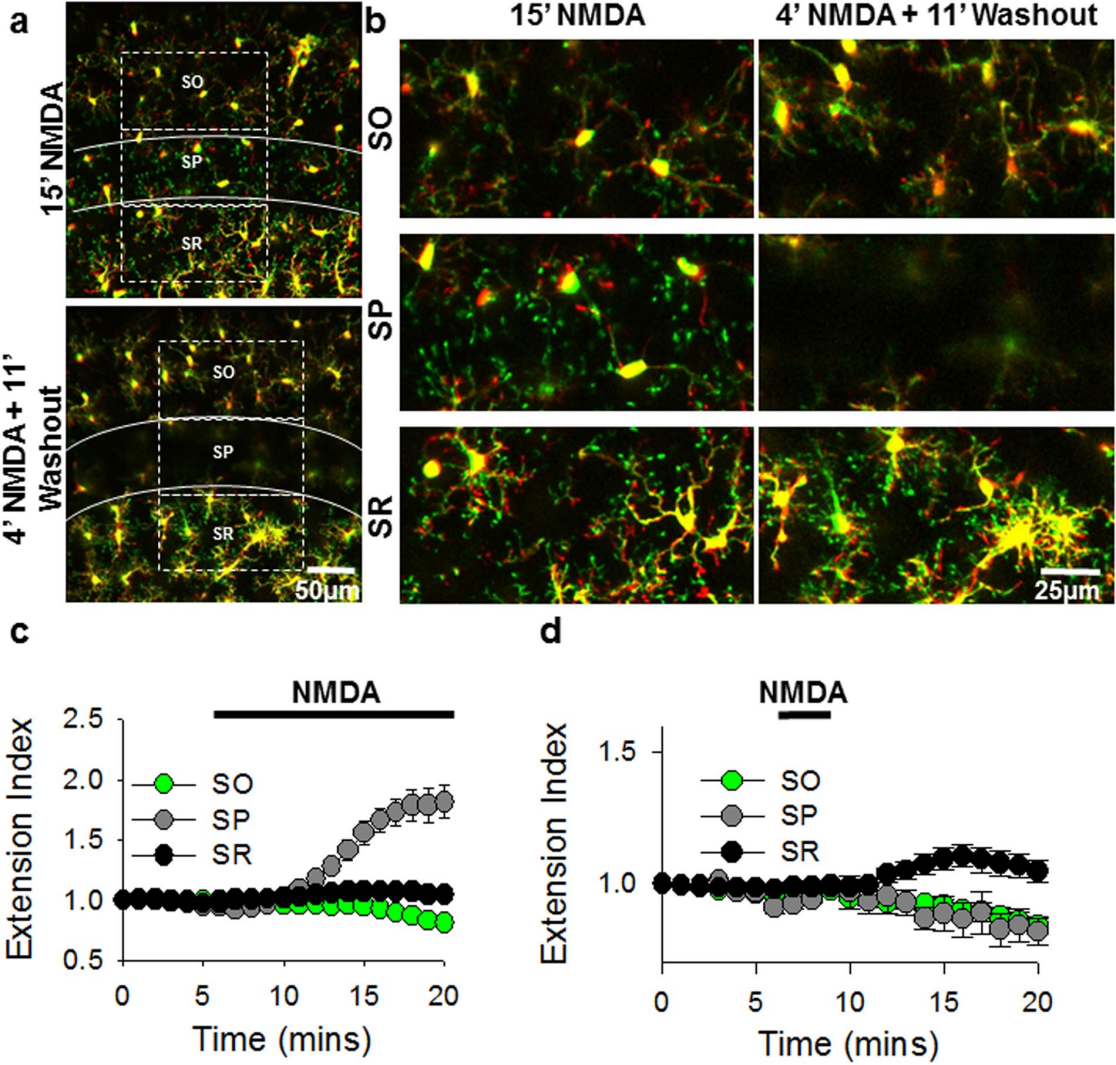
Differential Sensitivity in Somatic and Dendritic Responses during NMDAR-Induced Microglial Process Extension. (**a**) Representative two-photon z-stack merged color-coded images from 15 (top) and 4 (bottom) min NMDA (30 µM) treatment. Color code indicate microglial morphologies before (red) and after (green) NMDA treatment. (**b**) Higher magnification two-photon z-stack images of the *stratum oriens* (SO) *stratum pyramidale* (SP) and *stratum radiatum* (SR) regions of the corresponding 15 (left images) and 4 (right images) min NMDA treatment images from (**a**). (**c,d**) Quantitative summary of microglial process extension in the different regions during a 15 min (**c**) or a 4 min (**d**) NMDA-treatment protocol. All data are presented as mean ± S.E.M. n = 3–4 slices each.

Previously, MPEs were shown to require brief, repeated NMDAR activation in the *stratum radiatum* (SR) of the CA1^26^. However, in our prolonged perfusion of NMDA/glutamate paradigm, we found that MPEs only occurred after at least 5 minutes of global NMDA/glutamate application in the *stratum pyramidale* (SP) of the CA1^18^. To address this seeming discrepancy, we performed experiments in which NMDA (30 µM) was applied for 4 minutes followed by a washout of the drug. Interestingly, under these conditions, while we could elicit MPEs in the SR, MPEs were not detectable in the SP (Fig. 4; see also Supplementary Video 4). In general, MPEs were not obvious in the *stratum oriens* in either the 4 minute or 15 minute NMDA application paradigm (Fig. 4). These results thus suggest that there is a differential sensitivity to NMDAR-induced MPEs in the CA1 region. Since GluN2A subunits are predominantly localized to synaptic regions^27,34^ such as the SR over extrasynaptic regions such as the SP and SO of the CA1, these results are consistent with a role for the GluN2A subunit in the differential sensitivity of MPEs in the SR compared to the SP.

### Differential Regulation of Microglial Process Extension between the DG and CA1

**Figure 5:**
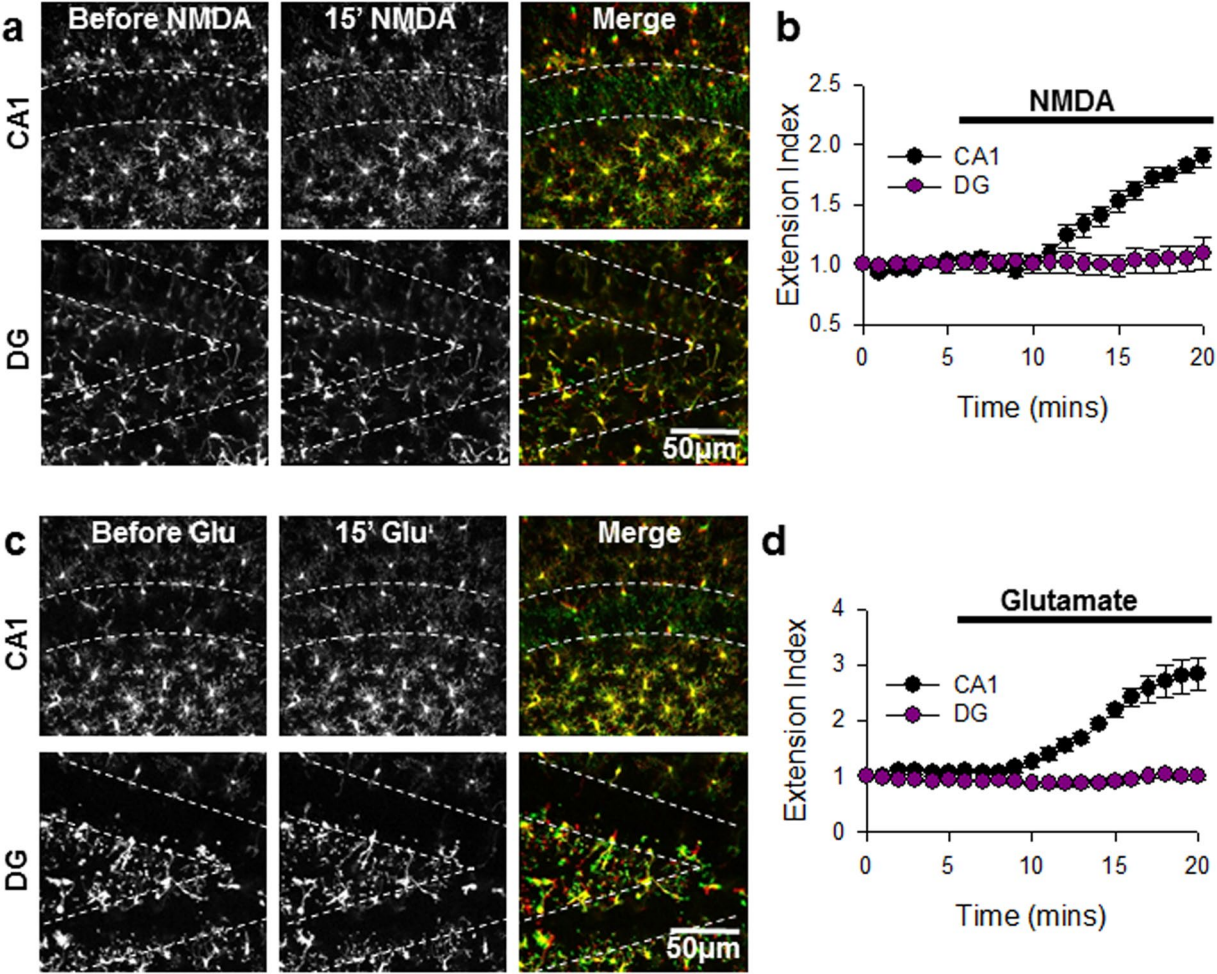
Regional Regulation of Microglial Process Extension. (**a**) Representative z-stack two-photon images of GFP-expressing microglia of acute brain slices before (left) and after 15 min of NMDA (30 µM) treatment (center) from the CA1 (top) and DG (bottom) regions of the hippocampus. Rightmost images are merged images of the before (red) and after (green) images. Extending microglial processes can be visualized in the neuronal cell body layer (dashed lines) in green. (**b**) Quantitative summary of corresponding data to (**a**); n = 4 slices each. (**c**) Representative z-stack two-photon images of GFP-expressing microglia of acute brain slices before (left) and after 15 min of glutamate (1 mM) treatment (center) from the CA1 (top) and dentate gyrus (DG, bottom) regions of the hippocampus. Rightmost images are merged images of the before (red) and after (green) images. Extending microglial processes can be visualized in the neuronal cell body layer (dashed lines) in green. (**d**) Quantitative summary of corresponding data to (**c**) All data are presented as mean ± S.E.M. n = 4–6 slices.

The GluN2A/GluN2B subunit ratio is higher in CA1 neurons compared to dentate gyrus (DG) neurons. Moreover, the GluN2A/B ratio remains unchanged in the DG while it is increased in the CA1 between neonatal development and adulthood^35^. On this basis, we speculated that DG neurons, given the maintenance of the immature GluN2A/GluN2B expression ratio even in adults, would fail to elicit MPEs. Indeed, upon the application of NMDA (30 µM), we detected a significant difference between the response of microglia in the DG and the CA1. Microglial processes exhibited a strong extension in the CA1 but lacked a response in the DG (extension index: 1.89 ± 0.08 in the CA1 and 1.1 ± 0.13 in the DG; Fig. 5a,b; See also Supplementary Video 5). Similar microglial responses were obtained with glutamate application (extension index: 2.83 ± 0.30 in CA1 and 0.99 ± 0.05 in the DG; Fig. 5c,d; see also Supplementary Video 6).

**Figure 6:**
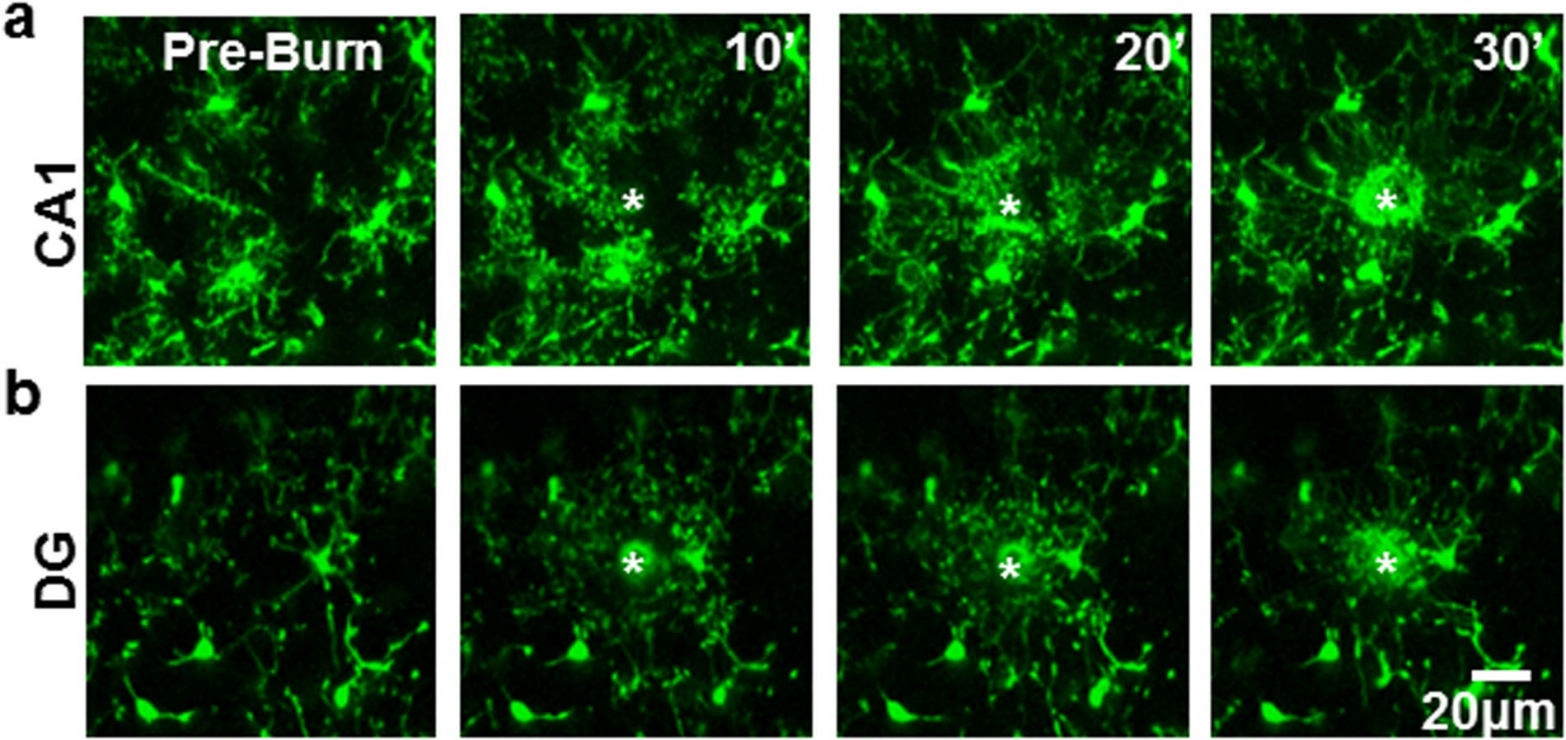
Microglial Chemotactic Responses are not Deficient in the Dentate Gyrus. (**a,b**) Microglial processes respond robustly to a laser-induced tissue injury (asterisks) by chemotaxis in both the CA1 (top panel) and the DG (bottom panel). n = 4 slices each.

The differences in the ability to elicit MPEs in the DG compared to the CA1 could be explained by one of the following possibilities. On the one hand, it is possible that neuronal NMDAR activation in the DG differs from that in the CA1. On the other hand, it is possible that microglial chemotactic responses to released ATP may differ between these two hippocampal regions. To rule out the possibility that DG microglia may lack chemotactic responses, a laser injury was induced in the CA1 (n = 5 slices) and DG (n = 4 slices) regions, and the extent and rate of microglial process extension to the injury were measured^36^. We found that microglia in both hippocampal regions are capable of responding robustly to laser-induced purinergic signals (Fig. 6; see also Supplementary Video 7). These results indicate that DG microglia are not deficient in their ability to respond to ATP. Together, our results indicate that the weaker MPE induction in the DG might result from differences in neuronal physiology rather than a microglial chemotactic function.

## Discussion

Microglial process extension (MPEs) and microglial process convergence (MPCs) in response to neuronal NMDAR activation was recently discovered in the murine brain^17,18,26^. In the current study, we show that GluN2A subunits of the NMDAR are critical for these microglia-neuron physical interaction phenomena. In this light, we show by proof of principle consistent with GluN2A roles that these interactions are (i) developmentally regulated, (ii) synaptically sensitive and (iii) differentially expressed in the CA1 and DG. These observations enhance our understanding of the mechanisms guiding physical microglia-neuron interactions that may be relevant during diseased contexts such as epilepsy and ischemic stroke.

Previous work had implicated NMDAR activation in the coupling of ATP release via pannexin channels during pathology^37^ and suggested this as a novel signaling modality for NMDARs^38^. Our findings here provide another possible mechanism that suggests the coupling of NMDAR activation to ATP release via the GluN2A subunit to elicit microglial process extension/convergence by P2Y12R activation. The precise avenue for ATP release remains to be determined, as previous work ruled out pannexin and connexin channels^17,18,26^. At the concentrations of glutamate/NMDA used, GluN2B subunits were also activated in slices. However, the lack of microglial morphological responses during GluN2B inhibition with its potent inhibitor (ifenprodil) suggest that the GluN2B subunits are not (or less) coupled to the ATP release pathway.

### Neuroprotective function of NMDAR-induced Microglia-Neuron Physical Interactions

We investigated the contribution of either the GluN2A or the GluN2B subunit to MPEs and found that the GluN2A receptor is predominantly responsible for NMDAR-induced MPEs/MPCs (Fig. 2). Emerging concepts (though without a full consensus) suggest that NMDARs with the GluN2A subunit are predominantly localized to the synapse, while those with the GluN2B subunit are predominantly extrasynaptic i.e. localized outside the synapse^27^. Similarly, studies suggest that synaptically localized GluN2A subunits mediate neuronal survival by inhibiting apoptotic mechanism mediated by synaptic calcium influx through these NMDARs. Conversely, extrasynaptic NMDARs with the GluN2B subunit are suggested to mediate neuronal demise through excessive calcium influx and pro-apoptotic mechanisms^39^. If this hypothetical framework is assumed for our studies and because we found a requirement for the GluN2A subunit of the NMDAR in MPEs, it is tempting to speculate that MPEs serve a neuroprotective function. This is consistent with our previous studies using P2Y12 and CX3CR1 knockout mice in the context of acute seizures where these receptors positively regulate these interactions^17,18,40^. There, we found that a genetic inhibition of MPEs and MPCs in these knockouts within an epileptic context correlated with a worsened behavioral phenotype during seizures. Since MPEs/MPCs require GluN2A activation, it is tempting to speculate that MPEs may serve neuroprotective functions.

### Developmental Regulation of NMDAR-Induced Microglia-Neuron Physical Interactions

We show that MPEs and MPCs are developmentally regulated since they are absent in immature neonatal tissues (Fig. 3). MPEs and MPCs continue to be detected from the second week of hippocampal development (data not shown) into adulthood^26^. Several microglia-neuron communication mechanisms are developmentally regulated. For example, microglial regulation of neurotransmission through CX3CR1 is present during early postnatal development but disappears in young mice in both the hippocampus^21^ and barrel cortex^20^. Moreover, microglial colonization of CNS tissues is also developmentally regulated through fractalkine signaling^20,21^.

In our study, the developmental regulation of MPEs and MPCs seems to be dependent on initiating neuronal mechanisms that require neuronal maturation. Microglia in neonatal tissues can respond robustly to purines through the engagement of their P2Y12 receptors^41,42^ whose mobilization is critical for MPEs^18,26^. This developmental regulation of MPEs/MPCs is consistent with the well-documented developmental shift from predominant GluN2B expression during early postnatal development to predominant GluN2A expression beginning in the second week of postnatal development^31–33^. Although we have not excluded other possibilities to be responsible for the differences observed with regards to MPEs/MPCs between neonatal and mature tissues, our data with regards to GluN2A NMDAR subunit roles is consistent with the observation of a developmental sensitivity to microglia-neuron physical interactions.

### Regional Regulation of NMDAR-induced Microglial Process Extension

One of the remarkable findings of this study is the differential regulation of MPEs in different brain regions. We found two levels of differential regulation: a localized differential regulation in the CA1 and a regionalized differential regulation between the CA1 and DG. Concerning the first level of regulation, we document a greater sensitivity to glutamate for MPEs in the *stratum radiatum* of the CA1 than the *stratum pyramidale*. This greater sensitivity for NMDAR-induced MPEs is consistent with GluN2A roles given that the subunit is predominantly localized to synaptic sites located in the *stratum radiatum*^27,39^. It is also possible that the ATP release mechanism may require a lower threshold for activation in this region than in the *stratum pyramidale*.

The second level of differential regulation of MPEs is between different regions of the hippocampus. Previously, we observed that MPEs occur in both the CA1 region of the hippocampus and layer II/III of the cortex^18,26^. However, we found that the phenomena are not present in all regions of the hippocampus as we could not elicit MPEs in the DG by either glutamate or NMDA application. We found that microglia resident in the DG were capable of functional chemotaxis in response to a laser-induced injury (Fig. 6b). Indeed, NMDA elicits differential release of neurotransmitters between the DG and CA1 e.g. for norepinephrine^43^ suggesting that DG neurons function differently than CA1 neurons to NMDAR activation. Consistently, DG neurons maintain an immature GluN2A/GluN2B expresssion ratio that does not change during development^35^ and DG express lower levels of GluN2A mRNA than in the CA1 of the adult rat^44^.

In summary, we have extended previous findings on microglia-neuron physical interactions in response to NMDAR activation and found its regulation predominantly through the GluN2A subunit of the NMDAR. Our study was performed in an entirely *ex vivo* slice system and raises questions as to the relevance of these findings *in vivo*, which will be addressed in future studies. Our previous^17,18^ and current results suggest that these interactions may serve a neuroprotective function, and the current findings provide novel insights into the mechanisms that may enhance our understanding of the dynamic interractions between microglia and neurons. Particularly, this neuroprotective interaction could be harnessed especially in conditions of excessive glutamatergic neuronal signalling such as seizures, epilepsy, and ischemic stroke.

## Methods

### Animals

Both male and female mice were used in accordance with guidelines of the Institutional Animal Care and Use Committee (IACUC) at Rutgers University and Mayo Clinic as approved by the Association for Assessment and Accreditation of Laboratory Animal Care (AAALAC) International. Heterozygous reporter mice expressing GFP under the control of the fractalkine receptor promoter (CX3CR1-GFP^+/−^)^45^ and YFP under the control of the Thy1 promoter^46,47^ were obtained from The Jackson Laboratory.

### Slice Preparation

Freshly isolated cortical or hippocampal slices were prepared from mice at various ages as stated in the results. Briefly, mice were anesthetized in an isofluorane (5%) chamber and swiftly decapitated. Brains from decapitated mice were carefully removed and placed in ice-cold, oxygenated (95%O_2_ and 5%CO_2_) artificial cerebrospinal fluid (ACSF) with the following composition (in mM): 124 NaCl, 25 NaHCO_3_, 2.5 KCl, 1 KH_2_PO_4_, 2 CaCl_2_, 2 MgSO_4_, 10 glucose, and sucrose added to make 300–320 mOsmol. Coronal slices (300 µm) were prepared and transferred to a recovery chamber for 30 or more minutes with oxygenated ACSF with the same composition as above at room temperature before imaging or electrophysiological studies.

### Whole cell patch Recording

Whole-cell patch recordings were performed in pyramidal neurons in live brain slices. The slices were placed in a stage and perfused with oxygenated (95% O_2_ and 5% CO_2_) artificial cerebrospinal fluid (ACSF) with the same composition as above. The recording pipette (3–5 MΩ) were filled with solution containing (mM): 115 Cs-MeSO3, 5 NaCl, 10 HEPES, 1 MgCl2, 0.2 EGTA, and 10 Na2Phosphocreatine (pH 7.2; 280–300 mOsmol). Data were amplified and filtered at 2 kHz by a patch-clamp amplifier (Multiclamp 700B), digitalized (DIGIDATA 1440 A), stored, and analyzed by pCLAMP (Molecular Devices, Union City, CA). NMDA (100 µM) was puff applied (10 psi, 10 ms, ~15 µm from patched cell) to induce EPSC. Access resistance of 15–30 MΩ was monitored throughout the experiment and data was discarded when changes more than 15% were observed. Antagonists were applied through the perfusion system.

### Two-photon Imaging

Experiments were conducted at room temperature with slices maintained in oxygenated ACSF with the same composition as above in a perfusion chamber at a flow rate of ~2 ml/min. Microglia were typically imaged using a two-photon microscope (Scientifica) with a Ti:sapphire laser (Mai Tai; Spectra Physics) tuned to 900 nm. Fluorescence was detected using two photomultiplier tubes in whole-field detection mode and a 565 nm dichroic mirror with 525/50 nm (green channel). The laser power was maintained at ~25 mW or below. We imaged microglia between 50 and 100 µm from the slice surface.

### Drugs

Glutamate, Ifenprodil and NMDA were purchased from Sigma. NVPAAM007 was a gift from Dr. Min Zhuo (University of Toronto). Stock solutions of all drugs were diluted to the working concentrations in ACSF and applied to the slices through a bath.

### Extension Index Analysis

Image analysis was done using ImageJ. Max projection images were collated to form time-lapse movies. Where necessary, movies were registered using the StackReg plugin to eliminate any *x*-*y* drift. For extension index, ROIs in the cell body layer of the CA1 or dentate gyrus (DG) were cropped from movies to be analyzed. These regions were then binarized and an automated threshold was set on the ROI. The area of the suprathresholded regions of the projection stack was then measured through time and normalized to the area of the first frame of the movie to a starting normalized index value of 1.0. The index through time of each time-lapse movie was then determined.

### Process Convergence Analysis

Quantification of microglial process convergence events was done manually as previously reported^17^. Events were identified when microglial processes spontaneously converged toward a focal point. To avoid arbitrary selection of these events, analysis was done by counting all the observed events irrespective of size so as not to bias our analysis/quantification. The frequency of occurrence of these events was determined in 330 × 330 × 45 µm field of view from 30-min long imaging sessions.

### Statistical Analysis

For all experimental analyses, 3–9 slices from different mice were analyzed and averaged to determine significance. Data are presented as mean ± SEM at the final recorded time point after drug application. Student’s T-test and one-way ANOVA with Bonferonni corrections where appropriate were used to establish significance.

## Electronic supplementary material

Supplemental Video 1

Supplemental Video 2

Supplemental Video 3

Supplemental Video 4

Supplemental Video 5

Supplemental Video 6

Supplemental Video 7

Supplementary File
