# Supplementary material for: The GluN2A Subunit Regulates Neuronal NMDA receptor-Induced Microglia-Neuron Physical Interactions
Source: Sci Rep. 2018 Jan 16;8:828. doi: 10.1038/s41598-018-19205-4 (PMC5770428; doi:10.1038/s41598-018-19205-4)
Supplement: Supplementary file 8 — Supplementary File [file 41598_2018_19205_MOESM8_ESM.docx]

**Supplementary Information**

**The GluN2A Subunit Regulates Neuronal NMDA receptor-Induced Microglia-Neuron Physical Interactions.**

Ukpong B. Eyo, Ashley Bispo, Junting Liu, Sruchika Sabu, Rong Wu, Victoria L. DiBona, Jiaying Zheng, Madhuvika Murugan, Huaye Zhang, Yamei Tang, Long-Jun Wu

**Supplemental Video 1:** A representative time lapse movie taken from CX3CR1^GFP/+^ mouse hippocampal slices before (10 min) and during (15 min) NMDA (30 µM) treatment with a GluN2B antagonist (Ifen; left) or a GluN2A subunit antagonist (NVP; right). This movie is 25 mins long.

**Supplemental Video 2**: A representative time lapse movie taken from CX3CR1^GFP/+^ mouse hippocampal slices before (5 min) and during (15 min) NMDA (30 µM) treatment in P7 (left) and one month old (right) tissues. This movie is 20 mins long.

**Supplemental Video 3:** A representative time lapse movie taken from CX3CR1^GFP/+^ mouse hippocampal slices before (5 min) and during (15 min) glutamate (1 mM) treatment in P7 (left) and one month old (right) tissues. This movie is 20 mins long.

**Supplemental Video 4:** A representative time lapse movie taken from CX3CR1^GFP/+^ mouse hippocampal slices before (5 min) and during 4 (left) or 15 min (right) NMDA (30 µM) treatment. The 4 min treatment elicits robust process extensions in the SR but not the SP, while the 15 min treatment elicits process extensions in both regions. This movie is 20 mins long.

**Supplemental Video 5:** A representative time lapse movie taken from CX3CR1^GFP/+^ mouse hippocampal slices before (5 min) and during (15 min) NMDA (30 µM) treatment in the CA1 (left) and the dentate gyrus (DG; right). Microglial process extensions occur in the CA1 but not in the DG. This movie is 20 mins long.

**Supplemental Video 6:** A representative time lapse movie taken from a CX3CR1^GFP/+^ mouse hippocampal slices before (5 min) and during (15 min) glutamate (1 mM) treatment in the CA1 (left) and the dentate gyrus (DG; right). Microglial process extensions occur in the CA1 but not in the DG. This movie is 20 mins long.

**Supplemental Video 7:** A representative time lapse movie taken from CX3CR1^GFP/+^ mouse hippocampal slices before (5 min) and following (20 min) a laser-induced tissue injury in the CA1 (left) and the dentate gyrus (DG; right). Microglial processes converge robustly towards the injury site in the center of the movie in both the CA1 and the DG. This movie is 25 mins long.
